# Physical Activity in the Prevention of Childhood Obesity: The Position of the European Childhood Obesity Group and the European Academy of Pediatrics

**DOI:** 10.3389/fped.2020.535705

**Published:** 2020-11-05

**Authors:** Justyna Wyszyńska, Susanne Ring-Dimitriou, David Thivel, Daniel Weghuber, Adamos Hadjipanayis, Zachi Grossman, Robert Ross-Russell, Katarzyna Dereń, Artur Mazur

**Affiliations:** ^1^Medical College of Rzeszów University, Rzeszów, Poland; ^2^European Childhood Obesity Group, Brussels, Belgium; ^3^Department of Sport and Exercise Science, Paris Lodron-University, Salzburg, Austria; ^4^Laboratory of the Metabolic Adaptations to Exercise Under Physiological and Pathological Conditions, Clermont Auvergne University, Clermont Ferrand, France; ^5^Department of Pediatrics, Paracelsus Medical University, Salzburg, Austria; ^6^Medical School, European University Cyprus, Nicosia, Cyprus; ^7^European Academy of Paediatrics, Brussels, Belgium; ^8^Adelson School of Medicine, Ariel University, Ariel, Israel; ^9^Maccabi Health Services, Tel Aviv, Israel; ^10^Department of Paediatric Respiratory Medicine, Cambridge University Hospitals National Health Service (NHS) Foundation Trust, Cambridge, United Kingdom

**Keywords:** adolescent, children, obesity, physical activity, recommendation

## Abstract

Obesity affects an increasing number of children and adolescents. Physical activity (PA) is a significant factor in the prevention of excessive body mass in the pediatric population. A significant percentage of pediatric population do not attain the public health recommendation for PA, and typically, those with higher levels of PA have lower content of body fat than less active peers. Although the development of childhood obesity is multifactorial, the decline in energy expenditure is considered as one of the most important determinants of excessive body weight. The lack of intervention causes that excess body weight to remain stable from birth through childhood and adolescence to adulthood. Accordingly, public health interventions are needed to increase the level of PA in the pediatric population. The task force from the European Childhood Obesity Group and the European Academy of Pediatrics reviewed English language meta-analyses, systematic reviews, randomized clinical trials, and observational studies from PubMed/MEDLINE, Cochrane Library, Science Direct, MEDLINE, and EBSCO databases, from 2018 to August 2020, and developed a consensus statement. This statement presents the role of PA in the prevention of excessive body weight and gives age-appropriate recommendations for PA and recommendations for school-based interventions, parents, and guardians.

## Introduction

Low level of physical activity (PA) contributes to ~5.3 million deaths a year and is a significant risk factor for non-communicable diseases (1). According to the World Health Organization (WHO), <20% of the world's adolescent population is sufficiently physically active (2). Evidence suggests that decreased level of PA is an important factor for the higher prevalence of childhood obesity (3). In 2016, it was estimated that more than 41 million children worldwide younger than 5 years were overweight (4). Overweight and obesity were diagnosed in more than 40% of children younger than 10 years in southern Europe, and fewer than 10% in northern Europe. Overall, the prevalence of overweight was more common in girls as compared with boys (21.1 vs. 18.6%) (5).

Although obesity is a complex, multifactorial disease, it mainly results from an imbalance between energy intake and energy expenditure. PA is the most modifiable factor of energy expenditure, accounting for about 25% of total energy expenditure, and as such, it is a powerful lever to affect the energy balance equation (6). Consequently, increasing the level of PA can contribute to improved weight management (7). Numerous cross-sectional studies demonstrate a negative association between the level of PA and overweight status in school-age children (8). Furthermore, evidence from the Finnish Twin Cohort study indicated that maintaining a higher level of PA is associated with lower body mass even after controlling for genetic factors and childhood environment. The study showed that cotwins with a higher level of PA have lower body mass, body mass index (BMI), and body fat, as well as they have much less visceral and hepatic fat than their less active cotwin (9).

## AIM

The aim of the statement was to present the role of PA in the prevention of excessive body weight and gives age-appropriate recommendations for PA and recommendations for school-based interventions, parents, and guardians.

## Methodology

Searching was conducted by using PubMed/MEDLINE, Cochrane Library, Science Direct, MEDLINE, and EBSCO databases, from 2018 to August 2020, for English language meta-analyses, systematic reviews, randomized clinical trials, and observational studies from all over the world. The websites of scientific organizations, such as WHO, were also searched. Selected articles were mutually agreed upon by the authors J. W. and A. M. Emphasis was given to the selection of meta-analyses and systematic review and information interesting for pediatric medical readership.

### PA Definition

PA is defined as “any bodily movement produced by the contraction of skeletal muscles that raises energy expenditure above the resting metabolic rate and is characterized by its modality, frequency, intensity, duration, and context of practice” (10, 11). PA is usually conceptualized as activity at least of light intensity (light-intensity PA 1.5–2.9 metabolic equivalent tasks, METs; 1 MET = 1 kcal kg^−1^ h^−1^) to positively stress the human body for structural and functional adaptations. However, especially in children, a health-enhancing PA dose is proposed that consists of moderate to vigorous (MVPA ≥ 3–5.9 METs) or even vigorous (VPA ≥ 6.0 METs) intensity (12). The term PA should not be mistaken with “exercise” that is defined as a subset of PA that is planned, structured, and repetitive and that favors physical fitness, maintenance, or development (10), as such activity can become part of a special exercise program in primary care or in therapy, compared to PA as an active lifestyle modality. Therefore, the term PA should not be confused with sports. Sport activities are a part of the PA and correspond to any institutionalized and organized form of movement in the sport system. Individuals engaged in sport activities are committed to specific rules during competitions and complete exercise training bouts with the purpose of improving their performance level. Accordingly, individuals engaged in lifestyle PA may train on a regular basis for the purpose of becoming healthier and/or to meet other people, but not necessarily to become sport athletes (11).

### The Importance of PA

Increasing the level of PA is associated with a wide spectrum of benefits, from improvements in lipid and glucose homeostasis to improved endothelial function. Such health outcomes have usually occurred independent of changes in BMI. A higher level of PA in childhood is correlated with lower risks of cardiovascular diseases, type 2 diabetes, and increased life expectancy in adulthood. The influence of PA on cardiovascular risk is associated with its effect on body fat (in particular abdominal fat) and insulin action. In this manner, exercise training improves capillarization and insulin sensitivity (13). The moderate intensity of PA contributes to achieving substantive health benefits. To achieve more favorable health effects, vigorous intensity activities are needed, with aerobic-based activities favoring greater health benefits (14). Maintaining a high level of PA is also associated with other numerous physical health benefits, including positive effects on body composition, blood pressure, metabolic profile, muscle growth, and bone mineral density (14–16). Results from a meta-analysis of 22 studies found that the risk of all-cause mortality was lower in subjects with a higher level of PA. It has been suggested that 2.5 h of moderate PA per week compared with no activity was related to a reduction in mortality risk of 19%, whereas 7 h a week reduced mortality risk by 24% (17). There is substantial evidence that being physically active has positive effects on psychosocial well-being, cognitive outcomes, and academic performance (e.g., grades and test scores), as well as mental health (18).

### Level of PA Among Children and Adolescents

Globally, ~20% of youth achieve the level of MVPA (60 min daily) recommended by the WHO (19), and in most European countries, fewer than 50% of children and adolescents meet these recommendations (20). However, there is large heterogeneity between countries. Collectively, evidence indicates that the level of PA decreases with age in adolescence, with girls being less active than boys (21). According to the Health Behavior in School-age Children (HBSC) survey, 23.1% of boys and 14.0% of girls aged 13–15 years accumulated 60 min of MVPA daily. Results from the HBSC survey comparing self-reported data on PA levels in the younger population in 36 European countries showed that the lowest proportion of children meeting the recommended 60 min of daily MVPA was observed in Italy (13%), Denmark (15%), and Greece (16%), whereas the highest percentage was in Finland (41%), Ireland (38%), and Bulgaria (36%) (22). According to the Active Healthy Kids Global Alliance, a substantial discrepancy in the amount of initiatives promoting PA and in the percentage of children reaching the recommended PA levels both within and across 38 countries from six continents (representing 60% of the world's population) was found (23). Denmark, Slovenia, and the Netherlands were the only countries with PA policies that facilitate the implementation of PA in daily life assessed by nine PA indicators (23). However, to establish accurate prevalence data on PA levels in youth, valid, and reliable measures are required. Data from the IDEFICS study, analyzing device-based measured PA levels using accelerometers among children aged from 2 to 10 years from eight European countries, showed that the percentage of individuals who were physically active for at least 60 min daily ranged from 2.0% (Cyprus) to 14.7% (Sweden) in girls and from 9.5% (Italy) to 34.1% (Belgium) in boys (24). Depending on the accelerometry cutoff point used to define MVPA, data show that at best at least 13%, and at worst 95–97%, of the young European population did not achieve the PA guidelines (25).

### Determinants of PA

Several factors specifically affecting children's PA participation have been identified. These determinants include individual (i.e., age and sex), sociocultural, and environmental factors. Evidence shows a prominent decline in PA with age, with an increase in sedentary behavior during adolescent years. Further, the age effect is mediated by gender, because a greater decline in PA was reported in girls compared to boys (26). Increased risk of insufficient levels of PA has been identified among children whose parents have lower education levels and children living in poverty, in apartments or public housing, and in neighborhoods where outdoor PA is limited by lack of facilities (27). Social factors such as the influence of parents had a significant impact on children's PA level. Parental PA, parental monitoring, and family support, particularly facilitation and encouraging or providing opportunities for PA of the child, have been found to significantly influence a child's activity (28). Younger adolescents appear to be especially affected by their same-sex parent. Moreover, support from peers also promotes PA in children (29), and there is evidence that there are positive correlations between self-efficacy level and PA (30). Environmental factors, including the facilities of school and community programs, physical environments, facility of fitness equipment at home, and exercise opportunities, are important determinants of PA in school-age children. Opportunities to participate in school physical education or organized sports are associated with greater time spent on MVPA. Physical safety is also considered as an important determinant of PA; high crime rates can promote inactivity (31, 32).

### Childhood Obesity Prevention Through PA

Preventing the development of childhood obesity is an international health priority. As obesity is a multifactorial disease, effective obesity prevention strategies should also target numerous aspects (personal, environmental, and socioeconomic). The first years of life are essential for starting preventive approaches that can have an impact on lifestyle and on developing overweight or obesity. In the case of young children, prevention strategies should concern parents, primary caregivers, schools, social networks, media, and the wider community (33). All of these groups should promote a healthy lifestyle with an appropriate level of PA and diet—either by providing an example to follow or by providing/favoring a supportive environment. Prevention programs should particularly involve the child's family. Parents should be a role model for a healthy lifestyle for their children. Because this parental practice is challenging, parents need social support to understand the importance of changing lifestyle habits and their role in the psychophysical development of their children (34).

For many children, increasing PA may be sufficient to prevent the onset of obesity. Healthy-weight children who are physically active tend to have less fat tissue than peers physically inactive. Therefore, a critical issue to maintain a healthy weight rests on the adoption of a suitable level of PA. Most European countries have developed national PA recommendations targeting children and adolescents (35); these recommendations are largely in accordance with the WHO recommendations. Amounts of PA greater than recommended minimum are associated with additional health benefits. A higher level of PA is associated with more favorable health parameters and improvements in health indicators, such as increased cardiorespiratory fitness and muscular strength, decreasing content of adipose tissue, improvement of cardiovascular disease risk profiles, and reduction of symptoms of depression (14, 36). PA should be adapted to children's physiological and metabolic profile; most of the daily PA should be aerobic (35, 36).

The home environment is a significant setting in preventing childhood obesity. Mainly, parents affect a child's health behaviors. Parents can influence children's PA through encouragement, involvement, and modeling (37, 38) Parental support, including encouragement and engaging with children in PA, is significant in maintaining a higher level of PA in children (39); however, findings indicated that this effect is inconsistent (40).

To prevent the growing obesity epidemic, a comprehensive policy approach influencing PA environments is also needed (41). Living in walkable neighborhoods, having parks, and good access to recreational facilities are associated with higher levels of PA and less likelihood of being overweight or obese (41).

As children spend a lot of time in schools, institutions of education represent an appropriate setting and opportunity to implement interventions that emphasize a healthy active lifestyle (42). In schools, there is an opportunity to increase children's PA levels, e.g., encouraging them to be active during break times or increasing the number of mandatory hours of physical education classes (43). Evidence suggests that a PA-friendly school environment is related to a lower risk of excessive body weight (44). Students in schools with at least three PA-friendly environmental factors (larger school campus size, more PA programs, better PA teaching experience, higher teachers' perceived PA benefits, more PA facilities, and better school PA ethos) had a much lower risk of prevalence of obesity than those without (44, 45). As a large number of preschool-age children spend much of their day in structured child care centers, there is also a need to promoting PA through these institutions (46). Evidence shows the associations between characteristics of the child care environment and children's PA. Large playgrounds with open space and access to portable play equipment (e.g., balls and hoops) were associated with a higher level of PA in preschool-age children. Policies promoting PA highlighted the need for teacher/staff training to implement structured PA programs (47).

Results from meta-analysis show that school-based interventions have positive effects on adiposity-related outcomes in 76 of 115 studies, but 42 of them were statistically significant. None of the home-based interventions reported statistically significant beneficial results (48). However, interventions that included both school and home elements had a significant effect on childhood obesity prevention (49–51). Evidence indicates that longer and multisetting, comprehensive school-based obesity interventions involving PA, diet, and health education are more effective than either a diet or PA intervention alone (48, 49, 52, 53). Educational institutions are attractive environments to promote positive health behaviors because they can cover a large number of children and overpass the potential barriers imposed by the children's “outside school” setting. Schools usually provide extracurricular activities. This is of particular importance because the timing of exercise and its impact on overall energy balance (energy expenditure and intake) have been recently highlighted, especially in the school setting (54, 55). There is also a need to emphasize the negative association of sugar-sweetened beverage consumption with child's health, not only in terms of direct impact on weight gain (56). Previous studies showed that soft drink consumption was inversely associated with PA level (57, 58). Moreover, energy drink consumption was associated with a higher risk of type 2 diabetes, heart disease, and stroke (59). Sugar-sweetened beverages should be replaced with other beverages, preferably water, to improve overall health. Thus, future interventions are needed to reduce sugar-sweetened beverages in the pediatric population, and the consumption of water and other non-sweetened beverages should be promoted also in the physical education context.

Numerous evidences reported an association between sedentary behavior (especially screen time) and overweight/obesity in children and adolescents (60). In fact, screen time competes with PA time and therefore displaces energy expenditure (61). In addition, screen time is associated with increased eating; exposure to high-calorie, low-nutrient food; and reduced sleep duration (62). Avon Longitudinal Study of Parents and Children found that sedentary behavior was positively associated with obesity in children, but this association was not independent of MVPA. Low levels of MVPA among the sedentary children increased the odds of obesity (63). A meta-analysis showed that reallocating sedentary time to MVPA was related to reduction in body fat content among youth (64). A recent meta-analysis indicated that screen time ≥2 h/day was associated with a higher risk of excessive body mass in children than screen time <2 h/day (odds ratio = 1.67). In order to prevent obesity in children and adolescents, lifestyle modification strategies should take into account screen time including total screen time, television/computer time, and smart phone use (65).

Evidence showed that children and adolescents with disabilities are significantly less active and more obese and experience more barriers to PA participation than those without disabilities (66). Barriers and facilitators to participate in PA must be better understood in order to implement effective interventions to prevent obesity in this population. Barriers included lack of knowledge and skills; the child's preferences; fear; parental behavior; negative attitudes to disability; inadequate facilities; lack of transport, programs, and staff capacity; and cost. The participation of children with severe disabilities in PA may also be impeded because of poor motor skills level and difficulties with social skills and communication, which limits opportunities to engage in team sports and other complex PA (67). Studies highlighted the necessity to provide the support from family, friends, and the community to encourage individuals with disabilities to participate in PA. Sports clubs should expand their programs and adapt them for those with disabilities. Moreover, information about PA programs for children with disabilities needs to reach parents, e.g., by improving collaboration between schools, sports clubs, and health care institutions. Family factors are significant determinants of participation children in PA. Parents who engage in PA themselves tend to promote similar participation for their children with disabilities. Health care providers should also support pediatric patients with disabilities by providing information about benefits from participation in PA and promoting effective interventions (68).

## Age-Appropriate Physical Activity Recommendations

Prevention interventions should start in the first years of life and continue through childhood, adolescence, and adulthood. This statement provides recommendations and guidelines for PA addressing children and adolescents, parents or legal guardians, or teacher and the school setting (35, 36, 69).

A task force from the Eastern Cooperative Oncology Group and the Extended Access Program, based on searched published literature, has agreed on the following recommendations for PA:

### Infants (<1 Year)

Infants (<1 year) should be supported to be physically active several times a day through supervised, interactive floor-based play; more is better. For infants who are not yet mobile, this contains at least 30 min of tummy time, spread throughout the day while awake. To achieve the PA recommendations, parents, or guardians should

- provide a safe and minimally structured play environment and
- engage infants in the unstructured exploration of the living and natural environment.

### Toddlers (1–2 Years)

Toddlers (1–2 years) should spend at least 180 min of PA at any intensity, including MVPA, spread throughout the day. More daily PA provides greater benefits.

To achieve the PA recommendations, parents or guardians should

- include a diversity of activities in different environments;
- include activities that develop movement skills;
- provide safe, nurturing, and both unstructured and minimally structured activity; and
- foster outdoor PA and the exploration of the natural environment (appropriate activities may include walking in the neighborhood, unorganized free play outdoors, play with a ball, or something similar).

### Pre-schoolers (3–4 Years)

Pre-schoolers (3–4 years) should spend at least 180 min in a variety of types of PA at any intensity, of which at least 60 min is MVPA, spread throughout the day. More daily PA provides greater benefits.

To achieve the PA recommendations parents, guardians or teachers should

- encourage engagement in a diversity of PA that support a natural development;
- encourage free play, with an emphasis on safe, enjoyable, and supervised motor tasks that promote playfulness and experimentation; and
- provide some structured play in a show-and-tell format to improve a broad spectrum of motor skills addressing balance (i.e., tumbling, dancing, and standing on one leg), locomotion (i.e., walking, running, jumping, swimming, and climbing), and object manipulation. (i.e., catching, throwing, and kicking).

### Children and Adolescents (5–18 Years)

Children and adolescents (5–18 years) should accumulate at least 60 min per day of MVPA involving a variety of aerobic activities. Amounts of PA >60 min daily provide additional health benefits. To achieve the PA recommendations, parents, guardians, or teachers should encourage children and adolescents to

- identify activities that are of their interest, as this is crucial for a long-term participation in PA;
- incorporate PA that promotes musculoskeletal and cardiovascular health at least 3 days per week (appropriate activities consist of playing games, running, swimming, dance, jumping, hopping, throwing, catching, etc.);
- incorporate active transportation and recreation, physical education, or planned exercise;
- engage in organized sports to learn a broad spectrum of different sports activities with a focus on social competence. For instance, sport games such as football provide insights into tactics and rules; and
- engage in supervised weight training to become aware of an appropriate technique and loading. Weight training with longer sets using heavier weights may be implemented as the adolescent reaches physical maturity (Tanner stage 5).

## Recommendations for Parents/Guardians

- Become a role model by maintaining an active lifestyle and participating in fun PA with children.
- Encourage and supervise mandatory MVPA duration.
- Provide enjoyable, age-, and performance-level appropriate activities (swimming, dancing, and sports) to foster the self-confidence of the child.
- Provide opportunities for child's PA to support the development of muscle strength and an increase in bone density.
- Enable children to participate in physical education in school, organized sport, and/or PA programs.
- Engage in school-led PA initiatives and continue these activities at home.
- Encourage friendships with active children.
- Encourage everyday activities and use active transportation, such as walking or cycling, use stairs instead of lifts/escalators, and get off bus a stop early.
- Limit sedentary behavior, particularly screen time to an age-appropriate cutoff (≤18 months: 0 min; pre-school children: <60 min; older children and adolescents: <120 min).
- Develop an active lifestyle–supportive environment for the child, such as no eating in front of the TV and removing the TV or screens from the child's bedroom.
- Create a safe indoor living environment with PA stimulating toys.
- Be a role model and stay active while watching TV by, for example, stretching, gymnastic exercises, or using exercise equipment. Encourage the child to join you while exercising in front of the TV.

## Recommendations for School-Based Interventions

Physical education programs should develop positive attitudes and motor and behavioral skills.

To achieve this guideline within and outside the school,

- increase the amount of mandatory physical education;
- encourage participation in physical education classes by all children regardless of disability;
- provide health education based on recommended PA levels within the classroom;
- promote PA during break and lunch periods;
- increase access to PA opportunities at school in addition to physical education;
- increase access to equipment that support PA (e.g., outdoor playgrounds);
- provide portable play equipment on playgrounds;
- increase time that children spend outside;
- reduce and partition children's sedentary time at school;
- promote the development of active and safe routes to school;
- increase the availability of safe and attractive, PA-stimulating playgrounds;
- improve collaboration between schools and families, local government, community recreation leaders, and health care professionals; and
- provide staff with training in the delivery of PA.

### Gaps and Implications

#### Individual Level

- To investigate the mediator role of PA on health behavior, i.e., how other behaviors related to childhood obesity are beneficially affected?
- To investigate the age-appropriate amount of PA on body composition
- To investigate the isotemporal (PA substitute to sedentary time) concept compared to the “addition” concept (PA increase and sedentary behavior reduction)

#### Interpersonal Level

- To investigate the impact of caregivers and/or peers on PA level of children and adolescents

#### Community Level

- To investigate the impact of the implementation of PA policies on the PA behavior (e.g., daily physical education in school)
- To investigate the setting specific impact of PA interventions on PA behavior (school setting vs. organized sport vs. informal sport/outside play vs. PA with family/peers)
- To develop effective strategies for increasing PA participation among children and adolescent with disabilities
- To develop effective strategies to improve participation in the programs and interventions
- To investigate effective strategies to promote PA in the childcare setting
- To conduct studies with longer intervention periods and long-term follow-up with rigorous measures.

## Conclusions

Childhood obesity is a multifactorial disease, influencing physical and mental health. Therefore, multidisciplinary, family-focused, and community-assisted strategies appear essential for the prevention of pediatric obesity in order to tackle the epidemic. The European Childhood Obesity Group and the European Academy of Pediatrics encourage health care professionals, teachers, parents, and guardians to promote PA to all children, from birth to adolescence.

## Author Contributions

JW, AM, DT, and SR-D were the project managers and conceived the manuscript design. DW, AH, and ZG participated in the design of the manuscript and collected data. JW, DT, SR-D, and KD analyzed the literature. JW drafted the manuscript. DT, SR-D, DW, AH, ZG, RR-R, KD, and AM revised the content of the manuscript. All authors contributed to the article and approved the submitted version.

## Conflict of Interest

The authors declare that the research was conducted in the absence of any commercial or financial relationships that could be construed as a potential conflict of interest.

## References

[1] Lee IM Shiroma EJ Lobelo F Puska P Blair SN Katzmarzyk PT Effect of physical inactivity on major non-communicable diseases worldwide: an analysis of burden of disease and life expectancy. Lancet. (2012) 9838:219–29. 10.1016/S0140-6736(12)61031-9 PMC3645500 22818936

[2] World Health Organization Prevalence of Insufficient Physical Activity. Global Health Observatory (2016). Available online at: http://www.who.int/gho/ncd/risk_factors/physical_activity/en/ (accessed September 20, 2019).

[3] Ng M Fleming T Robinson M Thomson B Graetz N Margono C. Global, regional, and national prevalence of overweight and obesity in children and adults during 1980–2013: a systematic analysis for the Global Burden of Disease Study 2013. Lancet. (2014) 9945:766–81. 10.1016/S0140-6736(14)60460-8 24880830 PMC4624264

[4] World Health Organization Facts and Figures on Childhood Obesity (2016). Available online at: http://www.who.int/end-childhood-obesity/facts/en/ (accessed September 20, 2019).

[5] Ahrens W Pigeot I Pohlabeln H De Henauw S Lissner L Molnár D Prevalence of overweight and obesity in European children below the age of 10. Int J Obes (Lond). (2014) 38(Suppl. 2):S99–107. 10.1038/ijo.2014.140 25376223

[6] Westerterp K Control of energy expenditure in humans. Eur J Clin Nutr. (2017) 71:340–344. 10.1038/ejcn.2016.237 27901037

[7] Swift DL Johannsen NM Lavie CJ Earnest CP Church TS The role of exercise and physical activity in weight loss and maintenance. Prog Cardiovasc Dis. (2014) 5:441–47. 10.1016/j.pcad.2013.09.012 PMC3925973 24438736

[8] Jiménez-Pavón D Kelly J Reilly JJ. Associations between objectively measured habitual physical activity and adiposity in children and adolescents: systematic review. Int J Pediatr Obes. (2010) 5:3–18. 10.3109/17477160903067601 19562608

[9] Piirtola M Kaprio J Waller K Heikkilä K Koskenvuo M Svedberg P. Leisure-time physical inactivity and association with body mass index: a Finnish Twin Study with a 35-year follow-up. Int J Epidemiol. (2017) 46:116–27. 10.1093/ije/dyw007 26979986

[10] Caspersen CJ Powell KE Christenson GM. Physical activity, exercise, and physical fitness: definitions and distinctions for health-related research. Public Health Rep. (1985) 100:126–31. 3920711 PMC1424733

[11] Thivel D Tremblay A Genin PM Panahi S Rivière D Duclos M. Physical activity, inactivity, and sedentary behaviors: definitions and implications in occupational health. Front Public Health. (2018) 6:288. 10.3389/fpubh.2018.00288 30345266 PMC6182813

[12] Tremblay MS Colley RC Saunders TJ Healy GN Owen N. Physiological and health implications of a sedentary lifestyle. Appl Physiol Nutr Metab. (2010) 35:725–40. 10.1139/H10-079 21164543

[13] Cesa CC Sbruzzi G Ribeiro RA Barbiero SM de Oliveira Petkowicz R Eibel B. Physical activity and cardiovascular risk factors in children: meta-analysis of randomized clinical trials. Prev Med. (2014) 69:54–62. 10.1016/j.ypmed.2014.08.014 25175591

[14] Janssen I Leblanc AG. Systematic review of the health benefits of physical activity and fitness in school-aged children and youth. Int J Behav Nutr Phys Act. (2010) 7:40. 10.1186/1479-5868-7-40 20459784 PMC2885312

[15] O'Malley G Ring-Dimitriou S Nowicka P Vania A Frelut ML Farpour-Lambert N. Physical activity and physical fitness in pediatric obesity: What are the first steps for clinicians? Expert conclusion from the 2016 ECOG workshop. Int J Exerc Sci. (2017) 10:487–96. 28674594 10.70252/YFYO2813 PMC5466409

[16] Fritz J Rosengren BE Dencker M Karlsson C Karlsson MK. A seven-year physical activity intervention for children increased gains in bone mass and muscle strength. Acta Paediatr. (2016) 105:1216–24. 10.1111/apa.13440 27096878

[17] Woodcock J Franco OH Orsini N Roberts I. Non-vigorous physical activity and all-cause mortality: systematic review and meta-analysis of cohort studies. Int J Epidemiol. (2011) 40:121–38. 10.1093/ije/dyq104 20630992

[18] Donnelly JE Hillman CH Castelli D Etnier JL Lee S Tomporowski P Physical activity, fitness, cognitive function, and academic achievement in children: a systematic review. Med Sci Sports Exerc. (2016) 48:1197–222. 10.1249/MSS.0000000000000901 27182986 PMC4874515

[19] Hallal PC Andersen LB Bull F Guthold R Haskell W Ekelund U. Global physical activity levels: surveillance progress, pitfalls, and prospects. Lancet. (2012) 380:247–57. 10.1016/S0140-6736(12)60646-1 22818937

[20] World Health Organization Physical Activity Factsheets For The 28 European Union Member States Of The Who European Region. (2018). Available online at: http://www.euro.who.int/__data/assets/pdf_file/0005/382334/28fs-physical-activity-euro-rep-eng.pdf?ua=1 (accessed September 21, 2019).

[21] Verloigne M Loyen A Van Hecke L Lakerveld J Hendriksen I De Bourdheaudhuij I. Variation in population levels of sedentary time in European children and adolescents according to cross-European studies: a systematic literature review within DEDIPAC. Int J Behav Nutr Phys Act. (2016) 13:69. 10.1186/s12966-016-0395-5 27350043 PMC4924322

[22] WHO Regional Office for Europe Growing Up Unequal: Gender and Socioeconomic Differences in Young People's Health and Well-being—Health Behaviour in School-aged Children (HBSC) Study: International Report from the 2013/2014 Survey Copenhagen. WHO Regional Office for Europe (2015). Available online at: https://www.euro.who.int/__data/assets/pdf_file/0003/303438/HSBC-No.7-Growing-up-unequal-Full-Report.pdf (accessed September 21, 2019).

[23] Tremblay MS Barnes JD González SA Katzmarzyk PT Onywera VO Reilly JJ Global Matrix 2.0: report card grades on the physical activity of children and youth comparing 38 countries. J Phys Act Health. (2016) 13(Suppl. 2):S343–66. 10.1123/jpah.2016-0594 27848745

[24] Konstabel K Veidebaum T Verbestel V Moreno LA Bammann K Tornaritis M Objectively measured physical activity in European children: the IDEFICS study. Int J Obes (Lond). (2014) 38(Suppl. 2):S135–43. 10.1038/ijo.2014.144 25376215

[25] Guinhouya BC Samouda H de Beaufort C. Level of physical activity among children and adolescents in Europe: a review of physical activity assessed objectively by accelerometry. Public Health. (2013) 127:301–11. 10.1016/j.puhe.2013.01.020 23582270

[26] Dumith SC Gigante DP Domingues MR Kohl HW III. Physical activity change during adolescence: a systematic review and a pooled analysis. Int J Epidemiol. (2011) 40:685–98. 10.1093/ije/dyq272 21245072

[27] Wilk P Clark AF Maltby A Smith C Tucker P Gilliland JA. Examining individual, interpersonal, and environmental influences on children's physical activity levels. SSM Popul Health. (2018) 4:76–85 10.1016/j.ssmph.2017.11.004 29349276 PMC5769121

[28] Hesketh KR O'Malley C Paes VM Moore H Summerbell C Ong KK. Determinants of change in physical activity in children 0–6 years of age: a systematic review of quantitative literature. Sports Med. (2017) 47:1349–74. 10.1007/s40279-016-0656-0 27988875 PMC5488114

[29] Yeung DC Yuan X Hui SS Feresu SA. Determinants of moderate to vigorous physical activity and obesity in children: a structural equation modeling analysis. World J Pediatr. (2016) 12:170–6. 10.1007/s12519-015-0057-8 26582296

[30] Cortis C Puggina A Pesce C Aleksovska K Buck C Burns C Psychological determinants of physical activity across the life course: a “DEterminants of DIet and Physical ACtivity” (DEDIPAC) umbrella systematic literature review. PLoS ONE. (2017) 12:e0182709 10.1371/journal.pone.0182709 28817676 PMC5560721

[31] Craggs C Corder K van Sluijs EM Griffin SJ. Determinants of change in physical activity in children and adolescents: a systematic review. Am J Prev Med. (2011) 40:645–58. 10.1016/j.amepre.2011.02.025 21565658 PMC3100507

[32] Oliveira AF Moreira C Abreu S Mota J Santos R Environmental determinants of physical activity in children: a systematic review. Arch Exerc Health Dis. (2014) 4:254–61. 10.5628/aehd.v4i2.158

[33] Han JC Lawlor DA Kimm SYS. Childhood obesity. Lancet. (2010) 375:1737–48. 10.1016/S0140-6736(10)60171-7 20451244 PMC3073855

[34] Watson PM Dugdill L Pickering K Bostock S Hargreaves J Staniford L. A whole family approach to childhood obesity management (GOALS): relationship between adult and child BMI change. Ann Hum Biol. (2011) 38:445–52. 10.3109/03014460.2011.590531 21682574

[35] Kahlmeier S Wijnhoven TMA Alpiger P Schweizer C Breda J Martin BW. National physical activity recommendations: systematic overview and analysis of the situation in European countries. BMC Public Health. (2015) 15:133. 10.1186/s12889-015-1412-3 25879680 PMC4404650

[36] World Health Organization Global Recommendations on Physical Activity for Health. Available online at: https://www.who.int/dietphysicalactivity/factsheet_recommendations/en/ (accessed September 26, 2019).

[37] Määttä S Ray C Roos E. Associations of parental influence and 10-11-year-old children's physical activity: Are they mediated by children's perceived competence and attraction to physical activity? Scand J Public Health. (2014) 42:45–51. 10.1177/1403494813504506 24026356

[38] Wilk P Clark AF Maltby A Tucker P Gilliland JA. Exploring the effect of parental influence on children's physical activity: the mediating role of children's perceptions of parental support. Prev Med. (2018) 106:79–85. 10.1016/j.ypmed.2017.10.018 29030264

[39] Yao CA Rhodes RE. Parental correlates in child and adolescent physical activity: a meta-analysis. Int J Behav Nutr Phys Act. (2015) 12:10. 10.1186/s12966-015-0163-y 25890040 PMC4363182

[40] Trost SG Loprinzi PD Parental influences on physical activity behavior in children and adolescents: a brief review. Am J Lifestyle Med. (2011) 5:171–81. 10.1177/1559827610387236

[41] Sacks G Swinburn B Lawrence M. Obesity policy action framework and analysis grids for a comprehensive policy approach to reducing obesity. Obes Rev. (2009) 10:76–86. 10.1111/j.1467-789X.2008.00524.x 18761640

[42] Gupta N Goel K Shah P Misra A. Childhood obesity in developing countries: epidemiology, determinants, and prevention. Endocr Rev. (2012) 33:48–70. 10.1210/er.2010-0028 22240243

[43] Powell E Woodfield LA Nevill AM. Increasing physical activity levels in primary school physical education: the SHARP principles model. Prev Med Rep. (2015) 3:7–13. 10.1016/j.pmedr.2015.11.007 26844179 PMC4733067

[44] Ip P Ho FK Louie LH Chung TW Cheung YF Lee SL. Childhood obesity and physical activity-friendly school environments. J Pediatr. (2017) 191:110–16. 10.1016/j.jpeds.2017.08.017 28987751

[45] Sallis JF McKenzie TL Beets MW Beighle A Erwin H Lee S. Physical education's role in public health: steps forward and backward over 20 years and HOPE for the future. Res Q Exerc Sport. (2012) 83:125–35. 10.1080/02701367.2012.10599842 22808697 PMC6036633

[46] European Commission/EACEA/Eurydice Key Data on Early Childhood Education and Care in Europe−2019 Edition. Eurydice Report. Publications Office of the European Union (2019).

[47] Ward DS Vaughn A McWilliams C Hales D. Interventions for increasing physical activity at child care. Med Sci Sports Exerc. (2010) 42:526–34. 10.1249/MSS.0b013e3181cea406 20068495

[48] Ickes MJ McMullen J Haider T Sharma M. Global school-based childhood obesity interventions: a review. Int J Environ Res Public Health. (2014) 11:8940–61. 10.3390/ijerph110908940 25170684 PMC4198999

[49] Wang Y Cai L Wu Y Wilson RF Weston C Fawole O. What childhood obesity prevention programmes work? A systematic review and meta-analysis. Obes Rev. (2015) 16:547–65. 10.1111/obr.12277 25893796 PMC4561621

[50] Bleich SN Vercammen KA Zatz LY Frelier JM Ebbeling CB Peeters A. Interventions to prevent global childhood overweight and obesity: a systematic review. Lancet Diabetes Endocrinol. (2018) 6:332–46. 10.1016/S2213-8587(17)30358-3 29066096

[51] Simon C Schweitzer B Oujaa M Wagner A Arveiler D Triby E. Successful overweight prevention in adolescents by increasing physical activity: a 4-year randomized controlled intervention. Int J Obes (Lond). (2008) 32:1489–98. 10.1038/ijo.2008.99 18626482

[52] Feng L Wei DM Lin ST Maddison R Ni Mhurchu C Jiang Y. Systematic review and meta-analysis of school-based obesity interventions in mainland China. PLoS ONE. (2017) 12:e0184704. 10.1371/journal.pone.0184704 28910362 PMC5598996

[53] Katz DL O'Connell M Njike VY Yeh MC Nawaz H. Strategies for the prevention and control of obesity in the school setting: systematic review and meta-analysis. Int J Obes (Lond). (2008) 32:1780–9. 10.1038/ijo.2008.158 19079319

[54] Reid RER Thivel D Mathieu ME. Understanding the potential contribution of a third “T” to FITT exercise prescription: the case of timing in exercise for obesity and cardiometabolic management in children. Appl Physiol Nutr Metab. (2019) 44:911–4. 10.1139/apnm-2018-0462 30875478

[55] Fillon A Mathieu ME Boirie Y Thivel D. Appetite control and exercise: Does the timing of exercise play a role? Physiol Behav. (2019) 218:112733. 10.1016/j.physbeh.2019.112733 31707067

[56] Dereń K Weghuber D Caroli M Koletzko B Thivel D Frelut M L. Consumption of sugar-sweetened beverages in paediatric age: a position paper of the European Academy of Paediatrics and the European Childhood Obesity Group. Ann Nutr Metab. (2019) 74:296–302. 10.1159/000499828 31013493

[57] Ranjit N Evans MH Byrd-Williams C Evans AE Hoelscher DM. Dietary and activity correlates of sugar-sweetened beverage consumption among adolescents. Pediatrics. (2010) 126:e754–61. 10.1542/peds.2010-1229 20876172 PMC3045775

[58] Park S Blanck HM Sherry B Brener N O'Toole T. Factors associated with sugar-sweetened beverage intake among United States high school students. J Nutr. (2012) 142:306–12. 10.3945/jn.111.148536 22223568 PMC4532336

[59] Scharf RJ DeBoer MD. Sugar-sweetened beverages and children's health. Annu Rev Public Health. (2016) 37:273–93. 10.1146/annurev-publhealth-032315-021528 26989829

[60] Biddle SJ García Bengoechea E Wiesner G. Sedentary behaviour and adiposity in youth: a systematic review of reviews and analysis of causality. Int J Behav Nutr Phys Act. (2017) 14:43. 10.1186/s12966-017-0497-8 28351363 PMC5371200

[61] Grgic J Dumuid D Bengoechea EG Shrestha N Bauman A Olds T. Health outcomes associated with reallocations of time between sleep, sedentary behaviour, and physical activity: a systematic scoping review of isotemporal substitution studies. Int J Behav Nutr Phys Act. (2018) 15:69. 10.1186/s12966-018-0691-3 30001713 PMC6043964

[62] Robinson TN Banda JA Hale L Shirong Lu A Fleming-Milici F. Screen media exposure and obesity in children and adolescents. Pediatrics. (2017) 140(Suppl. 2):S97–101. 10.1542/peds.2016-1758K 29093041 PMC5769928

[63] Mitchell JA Mattocks C Ness AR Leary SD Pate RR Dowda M. Sedentary behavior and obesity in a large cohort of children. Obesity (Silver Spring). (2009) 17:1596–602. 10.1038/oby.2009.42 19247272 PMC2746930

[64] García-Hermoso A Saavedra JM Ramírez-Vélez R Ekelund U Del Pozo-Cruz B Reallocating sedentary time to moderate-to-vigorous physical activity but not to light-intensity physical activity is effective to reduce adiposity among youths: a systematic review and meta-analysis. Obes Rev. (2017) 18:1088–95. 10.1111/obr.12552 28524399

[65] Fang K Mu M Liu K He Y. Screen time and childhood overweight/obesity: a systematic review and meta-analysis. Child Care Health Dev. (2019) 45:744–53. 10.1111/cch.12701 31270831

[66] Rimmer JA Rowland JL. Physical activity for youth with disabilities: a critical need in an underserved population. Dev Neurorehabil. (2008) 11:141–8. 10.1080/17518420701688649 18415819

[67] Shields N Synnot AJ Barr M. Perceived barriers and facilitators to physical activity for children with disability: a systematic review. Br J Sports Med. (2012) 46:989–97. 10.1136/bjsports-2011-090236 21948121

[68] Bloemen M Van Wely L Mollema J Dallmeijer A de Groot J. Evidence for increasing physical activity in children with physical disabilities: a systematic review. Dev Med Child Neurol. (2017) 59:1004–10. 10.1111/dmcn.13422 28374442

[69] World Health Organization Guidelines on Physical Activity, Sedentary Behaviour and Sleep for Children Under 5 Years of Age. WHO (2019). Available online at: https://apps.who.int/iris/bitstream/handle/10665/311664/9789241550536-eng.pdf?sequence=1&isAllowed=y (accessed May 2, 2019). 31091057

